# Supplementary figures and images for: A unitary mechanism underlies adaptation to both local and global environmental statistics in time perception
Source: PLoS Comput Biol. 2023 May 5;19(5):e1011116. doi: 10.1371/journal.pcbi.1011116 (PMC10191274; doi:10.1371/journal.pcbi.1011116)

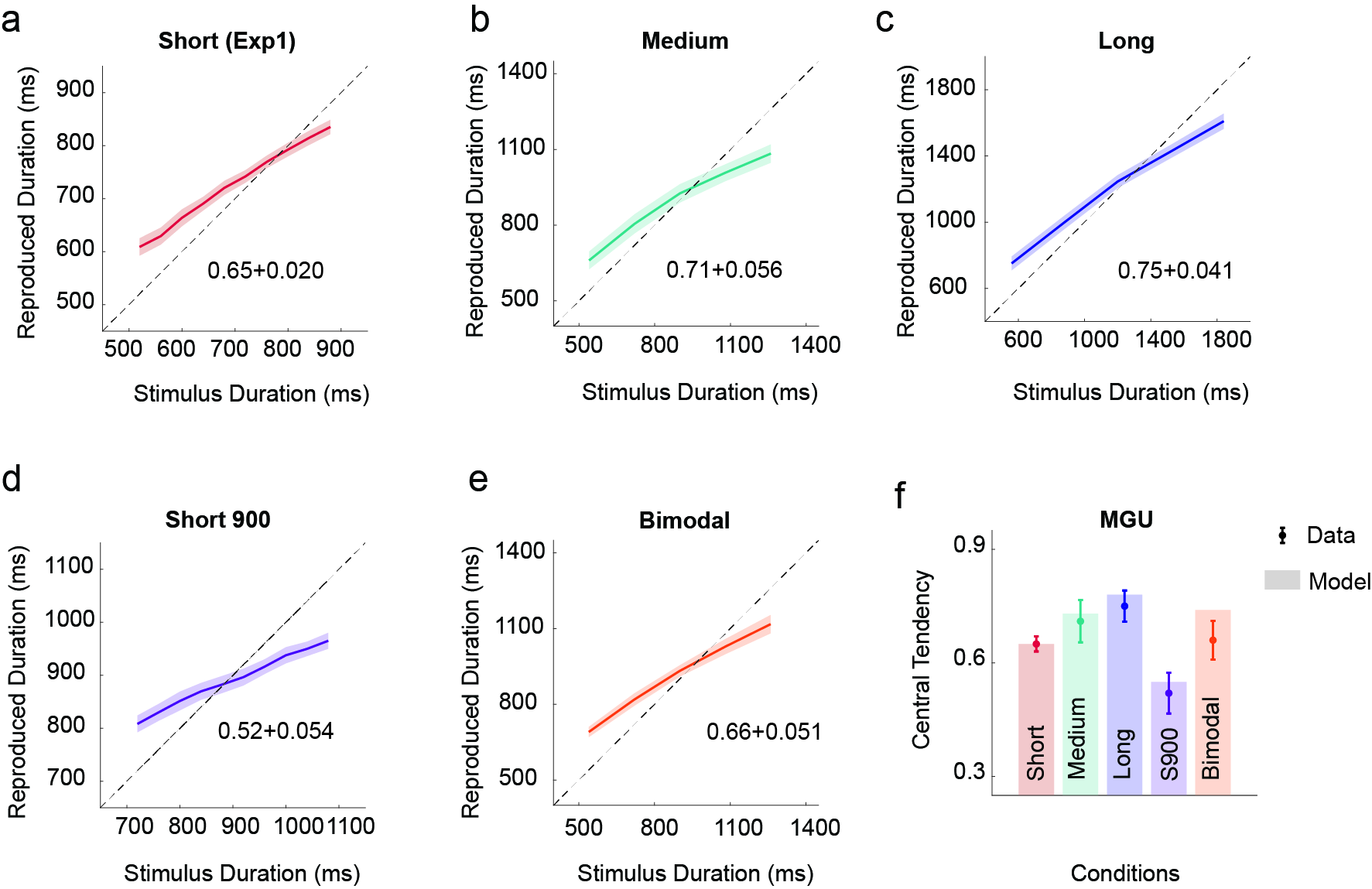

Supplement: S1 Fig — (a-e) Reproduced duration is plotted as a function of target duration for Experiments 1, 3, & 4. The shaded area indicates S.E. The median slope ± S.E. is reported on each figure. (f) The predicated slopes for the central tendency of the MGU model provide a good fit to the data. The dots and error bars indicate median slope ± S.E. (TIF) [file pcbi.1011116.s001.tif]

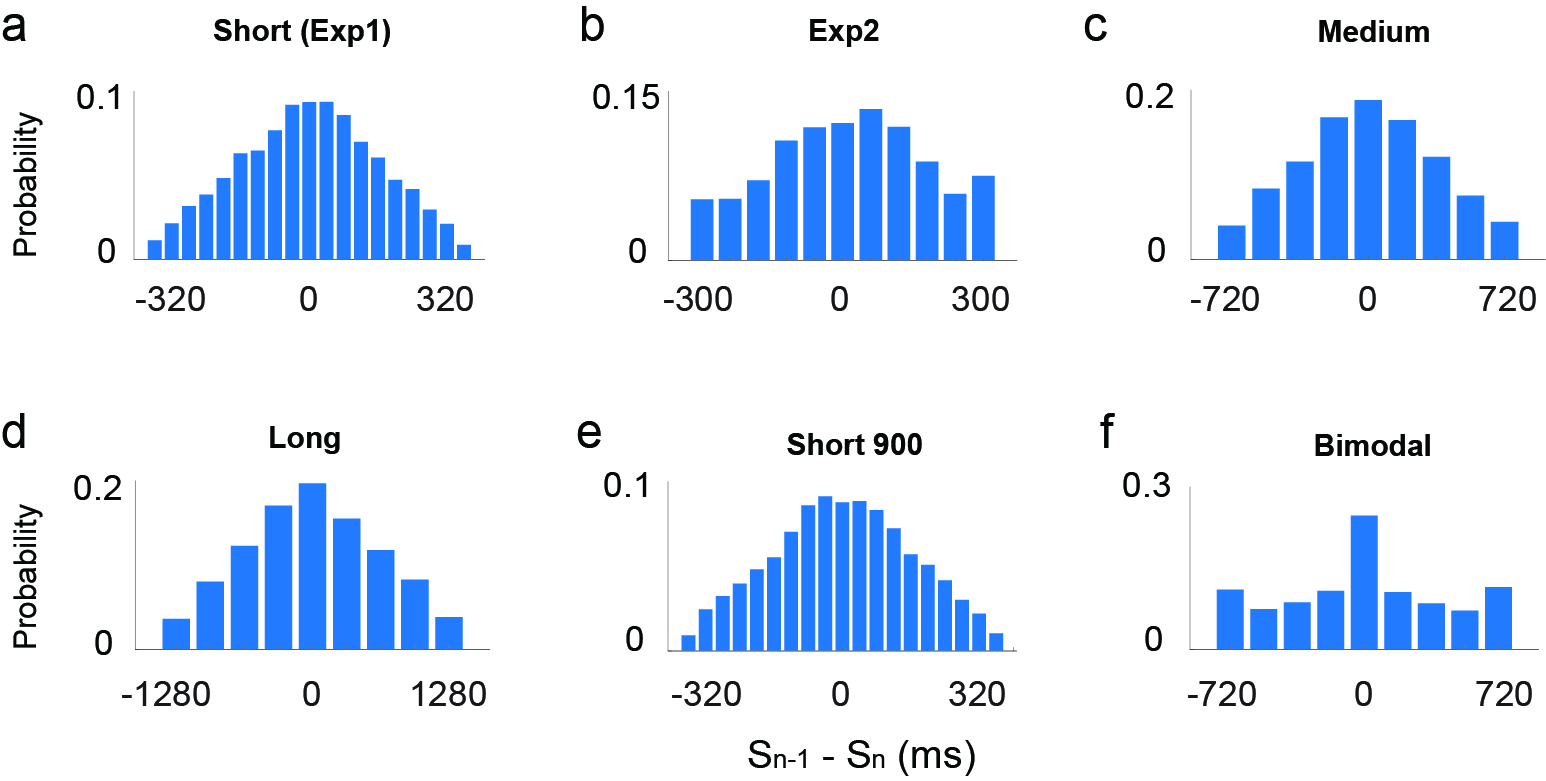

Supplement: S2 Fig — For individual participants, there are few trials with a large difference between stimuli, especially for experiment 1 and the short-900 condition. As such, the DoG may not provide a good fit when used to estimate serial dependence curves at the individual level. (TIF) [file pcbi.1011116.s002.tif]

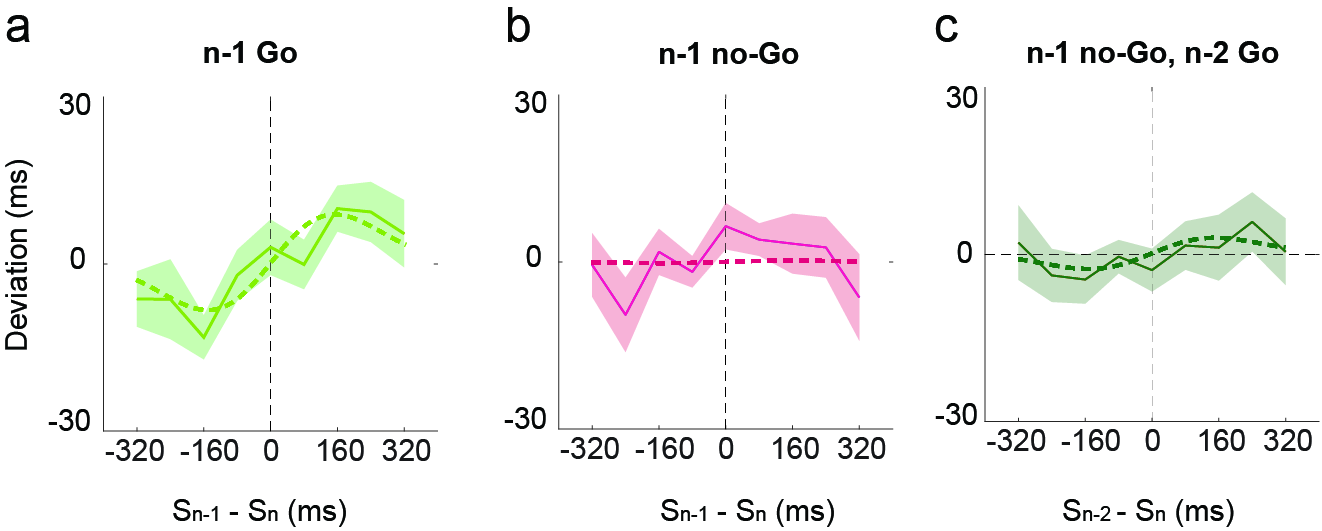

Supplement: S3 Fig — (a) A prominent DoG curve can be seen for the n-1 data when trial n-1 was a Go trial. (b) This curve is markedly attenuated when trial n-1 was a no-Go trial. (c) A small DoG curve is evident for the n-2 trial when n-1 was a No-Go trail and n-2 is a Go trial. The thick dashed line is the best-fitted DoG curve. Shaded areas indicate standard error. (TIF) [file pcbi.1011116.s003.tif]

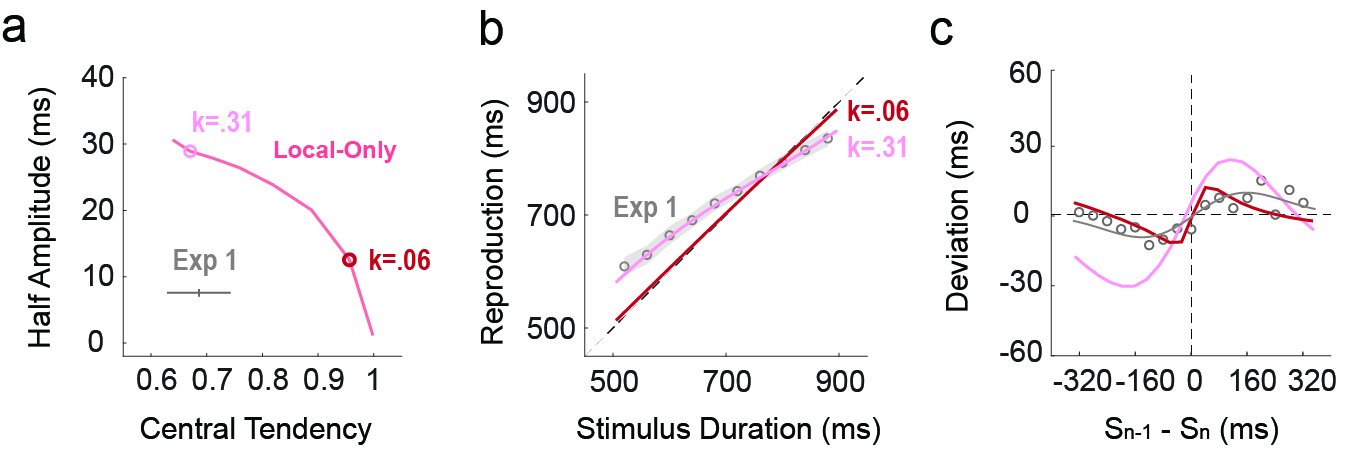

Supplement: S4 Fig — (a) Predicted relationship between central tendency and serial dependence for the local-only models. The depicted function shows the prediction of the model as the value of K is manipulated. The gray bar indicates the data from Exp 1, with the width of the bars indicating SE. (Note that the SE of half amplitude is very small). (b-c) Prediction of the central tendency and serial dependence effects for models with a K value of either 0.31 or 0.06. The model cannot capture both effects simultaneously. (TIF) [file pcbi.1011116.s004.tif]

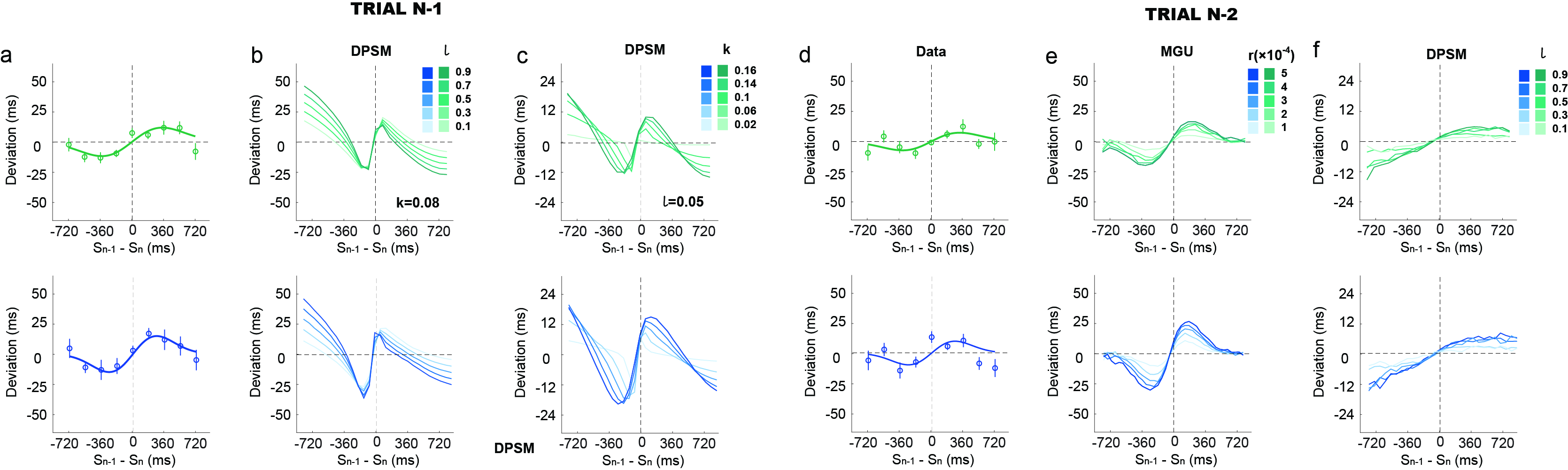

Supplement: S5 Fig — (a) Serial dependence function of the 1-back trial in the medium (top row) and long (bottom row) conditions of Exp 3. Thick line indicates the best-fitted DoG function. (b-c) 1-back serial dependence function predicted by the DPSM model with different values of the L (b) and k (c) parameters. In all simulations, the DPSM fails to generate a DoG-shaped serial dependence function for the 1-back trial. (d) Serial dependence function of the 2-back trial. Thick line indicates the best-fitted DoG function. (e-f) 2-back serial dependence function predicted by the MGU and DPSM models. The DPSM model predicts a monotonic, near-linear serial dependence function for the 2-back trial. Error bars indicate standard error. (TIF) [file pcbi.1011116.s005.tif]

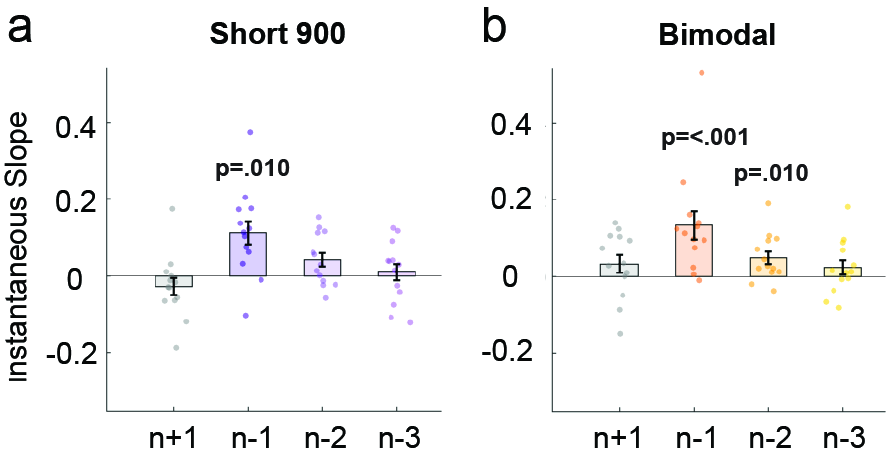

Supplement: S6 Fig — Instantaneous slope of the fitted DoG functions for trials n-1, n-2, and n-3, and future (n+1) trials in the short-900 (a) and bimodal conditions (b). A significant serial dependence effect can be observed from the n-2 trial in the bimodal condition rather than the short-900 condition in experiment 4. Dots indicate individual data points and error bars represent standard error. The p-values are based on a test of whether the observed values differ from zero. (TIF) [file pcbi.1011116.s006.tif]
