# Supplementary material for: A unitary mechanism underlies adaptation to both local and global environmental statistics in time perception
Source: PLoS Comput Biol. 2023 May 5;19(5):e1011116. doi: 10.1371/journal.pcbi.1011116 (PMC10191274; doi:10.1371/journal.pcbi.1011116)
Supplement: Attachment — Submitted filename: Reviewer_ploCB_5april2023.docx [file pcbi.1011116.s007.docx]

**Response Letter**

We thank the reviewers for the encouraging feedback. We are encouraged to see that the reviewers found our study interesting and thought-provoking.

We have thoroughly revised our paper based on the reviewers’ comments. The major changes are new modeling results and a detailed discussion about the potential limitations of our work. We believe these revisions have led to significant improvements in the paper and thank the reviewers for the thoughtful insights that have inspired these changes.

We provide a point-by-point reply to the reviewers' comments in the following sections. The reviewers' comments are presented in black text, while our responses are in blue. Additionally, we have highlighted all the changes we made in red within the manuscript file.

Thank you for your consideration of our revised manuscript. We look forward to the evaluation.

**Reviewer #1**

Major comments:

1. First and foremost, the serial dependence in duration perception has been reported (e.g., Togoli et al., 2021; Wiener et al., 2014; Wiener & Thompson, 2015), which should be acknowledged accordingly. Moreover, Zimmermann & Cicchini (2020) explicitly claims that such a contextual effect in time perception occurs at the perceptual level. The authors already discuss this perception vs. movement/response topic in a very balanced manner (ll.443-450), which is great, but mostly based on serial dependencies in other domains. I would suggest citing the papers above in the same paragraph.

Thanks for noting these relevant papers. We have now cited these papers in lines 472-473:

“We note that serial dependence effects have been observed in previous studies that did not involve motor responses, including judgments of duration (Wiener, Thompson and Coslett, 2014; Wiener and Thompson, 2015; Togoli *et al.*, 2021; Zimmermann and Cicchini, 12/2020) or visual orientation or position (Pascucci *et al.*, 2019; Fritsche, Spaak and de Lange, 2020; Manassi *et al.*, 12/2018).”

As suggested by Kiyonaga et al. (2017) and further supported by many subsequent studies, there are many forms of serial dependence. Serial dependencies are stimulus- and/or task- and/or modality-specific. Thus, as one possibility, the serial dependence induced by time reproduction may be specific to motor timing.

This is an important point—while it might be nice and tidy if there was one explanation for all serial dependence effects, we agree with the reviewer that there are many forms. We have revised the Discussion to acknowledge this point in lines 476-479:

“It is possible that the design used in Exp 2 may have been insensitive to capture a perceptual contribution to the serial dependence effect. Alternatively, the absence of a perceptual may be specific to the duration reproduction task used in our experiments. Indeed, there is evidence from duration comparison tasks without reproduction of an attractive sequential effect (Sierra, Poeppel and Tavano, 2021; Dyjas, Bausenhart and Ulrich, 11/2012). Follow-up experiments are needed to understand the factors that determine the weight given to perception and production in forming the prior. “

2. The models and figures require substantial modifications. Please see below for details.

2-1. Figure 3

The flow chart and color codes are somewhat complicated. Also it appears that the DP and Local-Only models yield the same prediction, which is actually not the case.

We have revised the arrangement of figure 3. For clarity, the figure is now separated into three columns. The left side illustrates how each model generates a posterior based on the current stimulus (likelihood). The middle column illustrates how motor output is decided based on the posterior, processes that are the same for all of the models. The right side illustrates the updating rules for the prior for the models.

“Figure 3. Schematics of the different models. (a) In all of the models, the target duration (green) is represented as a normal distribution (likelihood) centered at the target duration with perceptual noise. The global prior (blue) is based on the distribution of the target stimuli. The posterior (yellow) is obtained by multiplying the global prior by the likelihood. (b) The mean of the posterior is computed as a single-value estimate. The reproduced duration is sampled from a normal distribution centered on the estimate with motor noise. (c-d) In the BU and MGU models, the observer updates the prior based on the reproduced duration after the motor response. (c) For the MGU model, a scaled normal distribution centered at the reproduced duration is added to the old prior, and the new prior is normalized. (d) For the BU model, the prior is updated with a Kalman filter. (e) For the Local-Only model, the likelihood is integrated with the local prior in a non-linear manner, with the weight on the local prior increasing when the likelihood and local prior are similar and decreasing when they are dissimilar. In the DP model, the observer is assumed to hold two priors, one global and one local. After the posterior is computed based on the global prior and likelihood, the posterior is integrated with the local prior to generate a second posterior (posterior’). (f) In the second step of the DP and Local-Only models, the new local prior is fully determined by the motor response of the current trial. As with the other three models, the reproduced duration is drawn from a normal distribution centered at the mean of the posterior with motor noise.”

(Panel a) The "perception noise" should be rephrased into "sensory noise" or something similar, as the term "perception noise" has an assumption that sensory likelihood IS perception. That is, it assumes that such a Bayesian integration happens after perceptual processes, contradicting recent studies (e.g., Cicchini, Benedetto, Burr, 2021; Murai & Whitney, 2021; etc.).

We replaced "perception noise" with "sensory noise".

(Panel b) The term "motor noise" was confusing until reading the Models section. It first appeared to me as if the response was directly drawn from the posterior and the posterior width is called motor noise. Could the authors somehow modify the figure or elaborate in the legend so it reflects what they actually did (convert the posterior to a single value [eq.3-4] and then introduce a scalar motor noise component [eq.5])?

In revised Figure 3, we explicitly illustrate temporal “estimation” as the mean of the posterior. We also illustrate how motor noise is added to this estimation resulting in the produced interval. The graphical changes are accompanied by modifications to the caption (lines 188):

“The mean of the posterior is computed as a single-value estimate. The reproduced duration is sampled from a normal distribution centered on the estimate with motor noise.”

2-2. Figure 4

(Panel a) The Global-Only model predicts only a tiny central tendency compared to the observed effect size. However, many studies have successfully predicted the central tendency with this model, including Jazayeri & Shadlen's seminal work and many subsequent papers (e.g., Cicchini et al., 2014 JNS; Karaminis et al., 2016; Martin et al., 2017; Murai & Yotsumoto, 2018; etc.). This is presumably because np and nm values selected in the simulation (l.732) were not suitable for the present dataset.

In the original submission, we selected a range of perceptual noise (scaled by parameter *K* in the Local-only model) that can generate a serial dependence effect with a peak value close to that of the behavioral results. This does predict a central tendency effect that is smaller than observed in the data. The reviewer is correct that we can tune the *K* value to generate a stronger central tendency effect, one that better fits this feature of the data; However, *K* values in this range will predict a serial dependence effect that is much larger than what was observed in Exp 1 (See the figure below). As such, the global-only model cannot simultaneously capture the observed central tendency and serial dependence effects. We now make this explicit in the revision in lines 222-225. We also provide a new supplemental figure to illustrate the trade-off in the global-only model.

“However, this model cannot simultaneously capture the observed serial dependence and central tendency effects (Fig. 4a-c, Fig S4). Parameter values that generate the observed serial dependence effect produce a smaller than observed central tendency effect. Parameter values that generate the observed central tendency effect produce an unreasonably large serial dependence effect.”

“Figure S4. A local-only model cannot explain the results in Exp 1 across a range of parameter values. (a) Predicted relationship between central tendency and serial dependence for the local-only models. The depicted function shows the prediction of the model as the value of *K* is manipulated. The gray bar indicates the data from Exp 1, with the width of the bars indicating SE. (Note that the SE of half amplitude is very small). (b-c) Prediction of the central tendency and serial dependence effects for models with a *K* value of either 0.31 or 0.06. The model cannot capture both effects simultaneously.”

(Panel b) The Local-Only model and the DP model show quite a narrow feature tuning, indicating a similar problem in parameter selection.

The reviewer is correct that increasing sensory noise will increase the half-width (illustrated in Fig S4). However, as noted above, large sensory noise will also increase the half-amplitude of the SD function. Importantly, no parameters values for the Local-Only and DP model can fit the half-amplitude, half-width, and central tendency effects simultaneously.

(Panel d) The prior(n-1) suddenly appears without any explanations as a uniform distribution spanning over 500-1900 ms, which is totally different from stimulus distribution. I guess this is partly because otherwise the posterior would be completely flat in the right panel, but this visualization is very confusing. To be clear, I'm NOT against the concept itself. Just the visualization.

Sorry for the confusion. We used the prior of the long condition in Exp 3 to graphically contrast two scenarios, one with a small difference between successive durations and one with a large difference between successive durations. As the reviewer notes, if we used the prior from Exp 1 (500 ms – 900 ms), the components (prior/posterior/likelihood) would be quite close to each other and difficult to read.

We have added a sentence to the figure legend (lines 208):

“For illustration purposes, we depict the n-1 prior as a broad, uniform distribution ranging from 500-1900ms, the range used for the long condition in Exp 3”

2-3. Figure 5

This figure and underlying models should be overhauled. To be fair, I personally advocate the MGU, and agree with the conclusion itself. However, I'm not fully sure if the behavioral data in the present study is really able to distinguish the MGU and the DP.

We thank the reviewer for the detailed comments that follow on how to modify the models. We have implemented the alternative models suggested by the reviewer and describe the results below.

The most serious concern is that the DP has a very strong and arbitrary assumption that the local prior depends only on reproduction(n-1). If the local prior is derived from short/working memory trace from previous trials, as suggested by recent studies, the local prior could extend in time over a couple of trials. This should yield similar predictions about SD(n-2) as the MGU. Would it be possible to implement such a temporal component to the DP model? It might be better to drop the n-2 arguments and Figure 6 unless the authors present another point that MGU and DP would yield distinct predictions.

In our initial submission, we included two extreme situations of the local and global prior, with the former determined by the 1-back trial and the latter determined by the actual distribution of the test set.

Following the reviewer's suggestion, we implemented a more general version of this idea, the Dual-prior short-term memory (DPSM) model in which the updating of the local prior ($t_{prior}$) after each trial incorporates information from previous trials with diminishing weight:

$$t_{prior,i+1}={lt}_{r,i}+(1-l)t_{prior,i}$$

where $l$ is the learning rate of the prior. The DPSM does not capture the core features of the data. In particular, it predicts a near-linear serial dependence function for the 2-back trial and an unreasonable serial dependence function for the 1-back trial (See below).

We have opted to keep Figure 6 which presents the simpler version of the effect of working memory on local prior, the DP model. However, we describe the more general variant in the Results section of the main text (lines 329-337) and method (718-725), and have added a new supplemental figure to show these results (Fig S5):

“The DPSM model generates a serial dependence from the 2-back trial (Fig S5). However, the model predicts a near-linear serial dependence function, a prediction at odds with the data. Moreover, for the 1-back serial dependence function, the DPSM model predicts the direction of the serial dependence will flip when the current and previous stimuli are distinct from each other; this prediction is also not consistent with the data. In sum, adding a more graded working memory component to the dual prior model cannot account for the results of our experiments.”

Figure S5. A dual-prior model with short-term memory (DPSM) fails to predict the non-linear serial dependence. (a) Serial dependence function of the 1-back trial in the medium (top row) and long (bottom row) conditions of Exp 3. Thick line indicates the best-fitted DoG function. (b-c) 1-back serial dependence function predicted by the DPSM model with different values of the L (b) and k (c) parameters. In all simulations, the DPSM fails to generate a DoG-shaped serial dependence function for the 1-back trial. (d) Serial dependence function of the 2-back trial. Thick line indicates the best-fitted DoG function. (e-f) 2-back serial dependence function predicted by the MGU and DPSM models. The DPSM model predicts a monotonic, near-linear serial dependence function for the 2-back trial. Error bars indicate standard error.

Furthermore, could the authors elaborate why the global prior is first integrated with the likelihood and then the local prior in the DP model? My hunch is the opposite. Would the prediction change if the order is flipped?

We had reasoned that early, low-level perceptual processing might rely more on a global prior decided by long-term experience. In contrast, later-emerging decision-making processes or the motor output would be more likely to be influenced by recent experience. These assumptions seem consistent with the observation that tasks with a decision-making component tend to yield stronger serial dependence effects than more perceptual-oriented tasks (Cicchini, Anobile and Burr, 2014; Pascucci *et al.*, 2019; Manassi *et al.*, 12/2018). As such, in the dual processes model we first integrated the likelihood with the global prior and then the local prior.

Nonetheless, we examined whether the results would be similar if the order of the computations was reversed (i.e., likelihood x the local prior 🡪 the posterior x the global prior). We found that the key predictions were very similar to the original DP model: The reverse DP model predicts that the half-amplitude of the serial dependence function will increase as the range of the test set is extended (see Panel a below, response letter only), similar to the original ordering of the computations. However, the reversed DP model fails to predict that increasing the variance of the test set will enhance the serial dependence effect (see Panel b). It does not explain the large difference observed in the data between the medium-range uniform condition and the bimodal condition.

Figure 4, Panel d: I understand this as a computational concept, but obviously human observers cannot retain such a complex prior like the prior(n) in the short condition (see Acerbi et al., 2014). I suppose this will hold even with simpler prior shapes, though. The same logic is used for the variance argument (Figure 7), but it's less intuitive.

We respectfully disagree. We think it is possible for an observer to apply a combination of Gaussian distributions to approximate complex distributions. This method has the computational advantage of being amenable for representing arbitrary distributions. Moreover, there is some evidence to suggest that this is biologically feasible. Narain et al (2018) proposed a physiology model to explain how cerebellar granule cells with different tuning functions can learn a prior as the sum of Gaussians. This assumption also matches the empirical results from Acerbi et al (2014) where the authors showed that a prior formed by the sum of Gaussians provided the best fit to the data.

(Panel e; ll.277-279) The sensory likelihood should follow Weber's law both in MGU and DP (and any other models). Why should this predict the range-dependency of serial dependence only for DP?

The noise level does influence serial dependence in both DP and MGU. For the DP model, the serial dependence effect increases in the long-range condition solely because the noise level increases. In contrast, for the MGU model, serial dependence will increase because of both the increase in the noise level and the change in the prior distribution. This observation was a motivation for testing serial dependence using test sets with different variances but the same range (Exp 4). We have revised the discussion on this issue (lines 276-282):

“The MGU and DP models both predict that extending the range of the durations in the test set will enhance the trial n-1 serial dependence effect (Fig 5). In the DP, sensory noise scales with duration, a form of Weber's law. Because of this, the likelihood becomes relatively flat when the range is increased (Fig 5e). This will result in a greater influence of the previous production and, thus, a strengthening of the serial dependence effect (Fig. 5f-g). Sensory noise also influences the serial dependence effect in a similar way in the MGU. In addition, the MGU model postulates that the observer builds a concentrated prior when the range is limited (Fig 5d right)…”

In any case, the novelty and strength of this study is that no existing models (Global prior, Kalman filter, etc.) can simultaneously implement central tendency and serial dependence including its tuning properties. I'm not sure if it's a good idea to dip into the debate about single vs. multiple mechanisms (it's certainly a quite important issue though!).

Given the new modeling results, we believe that our study does offer value concerning the question of single vs multiple processes. Our results show that a single-process can provide a good account of the data. Nonetheless, we recognize that there could well be multiple-process models that also provide a good account of the data. We address this point in a new paragraph we have added to the Discussion (lines 439-448):

"We have presented evidence that a single prior which is dynamically updated across trials is sufficient to explain both the serial dependence and central tendency effects, as well as how they are jointly modulated by the distribution of the test set. Simple dual-prior models with a fixed global prior accompanied by a Gaussian-shaped or single-value prior determined by the most recent experience (1-back trial or short-term memory) cannot account for the behavioral results. Nonetheless, we cannot rule out that the pattern of results reported across the experiments can be captured by alternative multiple process models. One such possibility is a model that incorporates multiple processes, each adhering to the MGU model, but exhibiting distinct learning rates. However, on the grounds of parsimony, the single process MGU model provides a comprehensive account of the data, setting a point of comparison for future research.”

Minor comments:

3. (citation on l.43) Both the central tendency and the serial dependence typically happen between discrete events, as in the present study. While Fischer paper is in line with this, Manassi paper demonstrates a contextual effect WITHIN a continuous event, which is wildly different from the focus of this study. Although Manassi paper is an excellent manifestation of a new form of serial dependence, I believe there should be more suitable papers to cite here. I would suggest the papers listed in comment #1.

Thanks for this suggestion. We removed Manassi et al, 2022 and now cite the following at this point in the manuscript (Wiener, Thompson and Coslett, 2014; Wiener and Thompson, 2015; Togoli *et al.*, 2021; Zimmermann and Cicchini, 12/2020).

4. (citation on l.49) I would suggest citing another relevant paper about Bayesian calibration of timing perception (Miyazaki et al., 2006, Nat. Neurosci.).

We have added this citation.

5. (citation for Kalman-filter model) In addition to the Internal Reference model by Dyjas and colleagues, Kalman(-like) filter has been used to model serial dependence and central tendency (e.g., Cicchini et al. 2014; Taubert et al., 2016; Petzschner & Glasauer, 2011).

Thanks for the pointers. These are certainly relevant and we have added these citations when introducing the BU model (lines 236)

5. (l.142) "the serial dependence effect is about 8%" While a few papers have tried to quantify serial dependence by height/width, most readers would not understand what the authors mean without explanation about how and why they chose to calculate the effect size this way. From another perspective, considering that the mean stimulus duration was 700 ms, one would argue that the half-height of ~7ms is just about 1% of stimulus duration.

We agree that this can be a source of confusion. Since this type of quantification is not critical and can be confusing given different ways to do the calculation, we opted to delete the sentence.

6. (ll.156-159) In Figure S3, n-1 no-Go trials seem to show positive serial dependence. Although only the instantaneous slope result is indicated in Fig.2c, was the half height also not significant? Also, perhaps the x-axis label should be S(n-2) - S(n) for the rightmost panel.

The half-height is not significant for n-1 in the no-go condition. As can be seen in the best-fitted DoG curve (Fig S3b), the half height is right at 0. We have corrected the x-axis in Fig S3c.

7. Some typos: (l.249) "integrated with a static global prior, generating *serial dependence*" -> maybe central tendency? (l.683) "The formulas [11] band [13] "-> [7] and [9] (Figure 2c, tick numbers on y-axis) 1 -> 10; -1 -> -10

Thank you. We have corrected these errors.

**Reviewer #2:**

The question is of interest, the methods appear sound, experiments, modelling and model tests are carefully done.

We thank the reviewer for the encouraging comments.

The manuscript is not easy to read, but this is also due to the topic chosen. I have mainly one concern. In my view the authors should not talk about “unitary mechanism” vs. “dual prior” models. According to my understanding one crucial difference between the “unitary” MGU and the “dual” DP model is that the former stores local information about all previous trials, whereas the latter stores local information only about the last trial (plus global info). This point should be highlighted in the discussion, while the claim of a “unitary” vs. different mechanisms should be downplayed. Also, I am pretty sure that a “dual prior” model covering more of the immediately previous info in its local prior could fit the results as well as does the current MGU. Except for that I recommend acceptance.

The reviewer proposes that we consider a dual prior model with a local prior that is influenced by short-term memory rather than the more restrictive version we included in the original submission where the influence was limited to the 1-back trial. This is similar to an issue raised by Reviewer 1. We have pasted our response to Reviewer 1 here.

In our initial submission, we included two extreme situations of the local and global prior, with the former determined by the 1-back trial and the latter determined by the actual distribution of the test set.

Following the reviewer's suggestion, we implemented a more general version of this idea, the Dual-prior short-term memory (DPSM) model in which the updating of the local prior ($t_{prior}$) after each trial incorporates information from previous trials with diminishing weight:

$$t_{prior,i+1}={lt}_{r,i}+(1-l)t_{prior,i}$$

where $l$ is the learning rate of the prior. The DPSM does not capture the core features of the data. In particular, it predicts a near-linear serial dependence function for the 2-back trial and an unreasonable serial dependence function for the 1-back trial (See below).

We describe this more general variant of DP in the Results section of the main text (lines 329-337) and method (718-725), and have added a new supplemental figure to show these results (Fig S5):

“The DPSM model generates a serial dependence from the 2-back trial (Fig S5). However, the model predicts a near-linear serial dependence function, a prediction at odds with the data. Moreover, for the 1-back serial dependence function, the DPSM model predicts the direction of the serial dependence will flip when the current and previous stimuli are distinct from each other; this prediction is also not consistent with the data. In sum, adding a more graded working memory component to the dual prior model cannot account for the results of our experiments.”

Figure S5. A dual-prior model with short-term memory (DPSM) fails to predict the non-linear serial dependence. (a) Serial dependence function of the 1-back trial in the medium (top row) and long (bottom row) conditions of Exp 3. Thick line indicates the best-fitted DoG function. (b-c) 1-back serial dependence function predicted by the DPSM model with different values of the L (b) and k (c) parameters. In all simulations, the DPSM fails to generate a DoG-shaped serial dependence function for the 1-back trial. (c) Serial dependence function of the 2-back trial. Thick line indicates the best-fitted DoG function. (d-e) 2-back serial dependence function predicted by the MGU and DPSM models. The DPSM model predicts a monotonic, near-linear serial dependence function for the 2-back trial. Error bars indicate standard error.

Given the new modeling results, we believe that our study does offer value concerning the question of single vs multiple processes. Our results show that a single-process can provide a good account of the data. Nonetheless, we recognize that there could well be multiple-process models that also a provide a good account of the data. We address this point in a new paragraph we have added to the Discussion (lines 439-448):

"We have presented evidence that a single prior which is dynamically updated across trials is sufficient to explain both the serial dependence and central tendency effects, as well as how they are jointly modulated by the distribution of the test set. Simple dual-prior models with a fixed global prior accompanied by a Gaussian-shaped or single-value prior determined by the most recent experience (1-back trial or short-term memory) cannot account for the behavioral results. Nonetheless, we cannot rule out that the pattern of results reported across the experiments can be captured by alternative multiple process models. One such possibility is a model that incorporates multiple processes, each adhering to the MGU model, but exhibiting distinct learning rates. However, on the grounds of parsimony, the single process MGU model provides a comprehensive account of the data, setting a point of comparison for future research.”

“sense of passage of time”: This concept should not be combined with measuring duration reproduction, because in the timing literature different measures and results are associated with “passage of time” and “time estimation”. So, please do not use this phrase in the present context.

Thank you for pointing out this distinction. We have modified the phrasing, changed “sense of passage of time” to “duration estimation”

Fig. 2 C, There are mistakes in the y-axis labels.

This has been corrected.

**Reviewer #3:**

Major comments:

I believe the authors should be careful with arguing that serial dependence and central tendency originate from the same mechanism. First, showing that a model can account for both effect does not necessarily implies a common mechanism. Second, and more importantly, serial dependence has some very peculiar characteristics (tunings) which cannot be explained by simple central tendency biases. For example, spatial tuning (ref 1-3) implies a difference in serial dependence independently on response reproduction or decision. In addition, serial dependence is gated by attention (ref 1). Can the unitary model explain these characteristics of serial dependence? The authors should test whether their model can account for the tuning properties of serial dependence.

While acknowledging that we may have misunderstood the reveiwer’s comment here, we offer two responses. The first concerns the question of whether serial dependence effects on spatial and temporal tasks have similar features (and thus might be amenable to a common modeling framework). The second is related to the issue single vs multiple mechanisms. We address each in turn.

**Commonality across task domains:** We think serial dependence and central tendency exhibit similar tuning properties. In terms of spatial turning, a target locates farther away from the 1-back target showed a weaker serial dependence effect(Fischer and Whitney, 2014). Similarly, in duration perception, participants can construct separate internal priors for stimuli that are presented at two different positions (Roach *et al.*, 2017). In both cases, the system relies less on the prior(s) when the stimulus changes position.

In terms of attention, an unattended stimulus did not induce a serial dependence effect in an orientation judgment task (Fischer and Whitney, 2014). Similarly, when participants are told in advance that they won’t need to reproduce a duration (so presumably this duration is unattended), the duration of the unattended stimulus does not influence the central tendency effect (Roach *et al.*, 2017). As such, we believe these cross-domain similarities are consistent with our single prior model.

Moreover, we see spatial and attentional tuning to be more about the learning and utilization of how prior(s) generalize across different contexts. We expect that the DP and MGU could predict these effects by adding specific generalization terms. However, we have opted to not address this point in the revised manuscript, feeling it is beyond the scope of the paper.

**Single vs multiple mechanisms:** This issue was also raised by Reviewers 1 and 2. We agree with the reviewer that our modeling results cannot rule out the possibility that some kinds of multi-processes models could explain the data. We address this point in a new paragraph we have added to the Discussion (lines 439-448):

"We have presented evidence that a single prior which is dynamically updated across trials is sufficient to explain both the serial dependence and central tendency effects, as well as how they are jointly modulated by the distribution of the test set. Simple dual-prior models with a fixed global prior accompanied by a Gaussian-shaped or single-value prior determined by the most recent experience (1-back trial or short-term memory) cannot account for the behavioral results. Nonetheless, we cannot rule out that the pattern of results reported across the experiments can be captured by alternative multiple process models. One such possibility is a model that incorporates multiple processes, each adhering to the MGU model, but exhibiting distinct learning rates. However, on the grounds of parsimony, the single process MGU model provides a comprehensive account of the data, setting a point of comparison for future research.”

Regarding Experiment 2, there is a conflicting literature on the role of previous decision/attention on serial dependence. Some studies have found that serial dependence does not necessarily depend on decision (ref 1,4), whereas others did (see Pascucci et al. 2019). I saw the authors acknowledged some of these findings, but it would be useful to rephrase statements like "This dissociation suggests that serial dependence in timing mainly arises from sequential effects associated with temporal reproduction". Several factors may be at play here, such as attention, decision, memory, motor response etc etc.

We recognize that the serial dependence effect may come about from one or more processes involved in temporal reproduction (e.g., attention, decision, motor response). To clarify this, we have revised the passage as follows (lines 159-162):

“This dissociation supports the idea that serial dependence in timing largely arises from factors associated with temporal reproduction. We recognize that this may reflect how reproduction is influenced by attention, decision making, memory, or the response itself."

Furthermore, we acknowledge in the Discussion that serial dependence could arise from perceptual processes, but our method may not be sufficiently sensitive to capture these effects (see lines 472-480).

Minor comments:

Line 76: "the serial dependence effect has not been well established in timing." There is a large body of literature of serial dependence in time perception (see 5-10 below). The authors should rephrase.

We have deleted this statement. We also include the literature of serial dependence in duration perception in the introduction (lines 39-43):

“Participants’ perception of the current duration is attracted toward the duration of the previous stimulus (Bausenhart, Bratzke and Ulrich, 04/2016; Bausenhart, Dyjas and Ulrich, 03/2014; Dyjas, Bausenhart and Ulrich, 11/2012). That is, the duration is perceived to be longer after a relatively long duration stimulus compared to a relatively short duration stimulus(Wiener, Thompson and Coslett, 2014; Wiener and Thompson, 2015; Togoli *et al.*, 2021; Zimmermann and Cicchini, 12/2020). This phenomenon, known as serial dependence, suggests that the perceptual system is also sensitive to the local statistics of the environment (Fischer and Whitney, 5/2014; Kiyonaga *et al.*, 07/2017).”

Figure 2 (and across the manuscript). The authors refer to "height" as the half amplitude of the DoG curve. Across the literature, this measure is called "half amplitude". It would be great if the authors could rephrase this to be consistent with the literature.

Thanks for pointing this out. We have replaced half height with half amplitude throughout the manuscript.
